# The Temporal Courses of Phonological and Orthographic Encoding in Handwritten Production in Chinese: An ERP Study

**DOI:** 10.3389/fnhum.2016.00417

**Published:** 2016-08-24

**Authors:** Qingfang Zhang, Cheng Wang

**Affiliations:** ^1^Department of Psychology, Renmin University of ChinaBeijing, China; ^2^Key Laboratory of Behavioral Science, Institute of Psychology, Chinese Academy of SciencesBeijing, China; ^3^College of Education, Zhejiang University of TechnologyHangzhou, China

**Keywords:** handwritten production, picture-word interference task, orthographic autonomy hypothesis, orthographic facilitation effect, phonological facilitation effect

## Abstract

A central issue in written production concerns how phonological codes influence the output of orthographic codes. We used a picture-word interference paradigm combined with the event-related potential technique to investigate the temporal courses of phonological and orthographic activation and their interplay in Chinese writing. Distractors were orthographically related, phonologically related, orthographically plus phonologically related, or unrelated to picture names. The behavioral results replicated the classic facilitation effect for all three types of relatedness. The ERP results indicated an orthographic effect in the time window of 370–500 ms (onset latency: 370 ms), a phonological effect in the time window of 460–500 ms (onset latency: 464 ms), and an additive pattern of both effects in both time windows, thus indicating that orthographic codes were accessed earlier than, and independent of, phonological codes in written production. The orthographic activation originates from the semantic system, whereas the phonological effect results from the activation spreading from the orthographic lexicon to the phonological lexicon. These findings substantially strengthen the existing evidence that shows that access to orthographic codes is not mediated by phonological information, and they provide important support for the orthographic autonomy hypothesis.

## Introduction

While a considerable amount of research has been conducted to identify the processes and mechanisms underlying spoken word production, relatively less research has addressed the process of handwritten word production. In the present study, we investigate lexical access, that is, the core process involved in the production of the spoken and written word with respect to written picture naming, which is a typical task employed in the production of the spoken word. The current models of speech production provide a general theoretical framework from which the hypothesis of writing can be derived. Early theorists lacked interest in writing and traditionally assumed that written production is parasitic on speech (Geschwind, 1969). This assumption raised a central debate in the field, i.e., whether orthographic output is constrained by phonological codes. According to the obligatory phonological mediation hypothesis (Geschwind, 1969; Luria, 1970), access to orthographic codes depends on prior phonological activation. This hypothesis was initially based on the premise that writing is usually accompanied by inner speech (Hotopf, 1980), and it gained support from phonologically based spelling errors (e.g., *there* spelled as *their, yacht* spelled as *yot*; Aitchison and Todd, 1982; Behrmann and Bub, 1992) and findings of comparable impairments in the spoken and written productions of a graphic patients (Luria, 1966; Basso et al., 1978).

This view is severely challenged by the neuropsychological findings of double dissociation between speaking and writing, thus indicating that orthographic activation does not depend on phonology. For example, some patients with brain damage were often able to write picture names but were unable to verbally name them (Bub and Kertesz, 1982; Rapp et al., 1997), while other patients, when presented with the same picture, produced inconsistent spoken and written responses (e.g., for a picture of *pliers*, they say *pincers* but write *saw*; Miceli et al., 1997; Alario et al., 2003). These types of findings support the orthographic autonomy hypothesis, which states that orthography can be accessed directly from semantic representations with no need for phonological involvement (Rapp et al., 1997). This account is further supported by behavioral studies on normal subjects that found that phonological overlapping did not facilitate written production (Bonin et al., 1998; Roux and Bonin, 2012; Shen et al., 2013).

**Figure 1:**
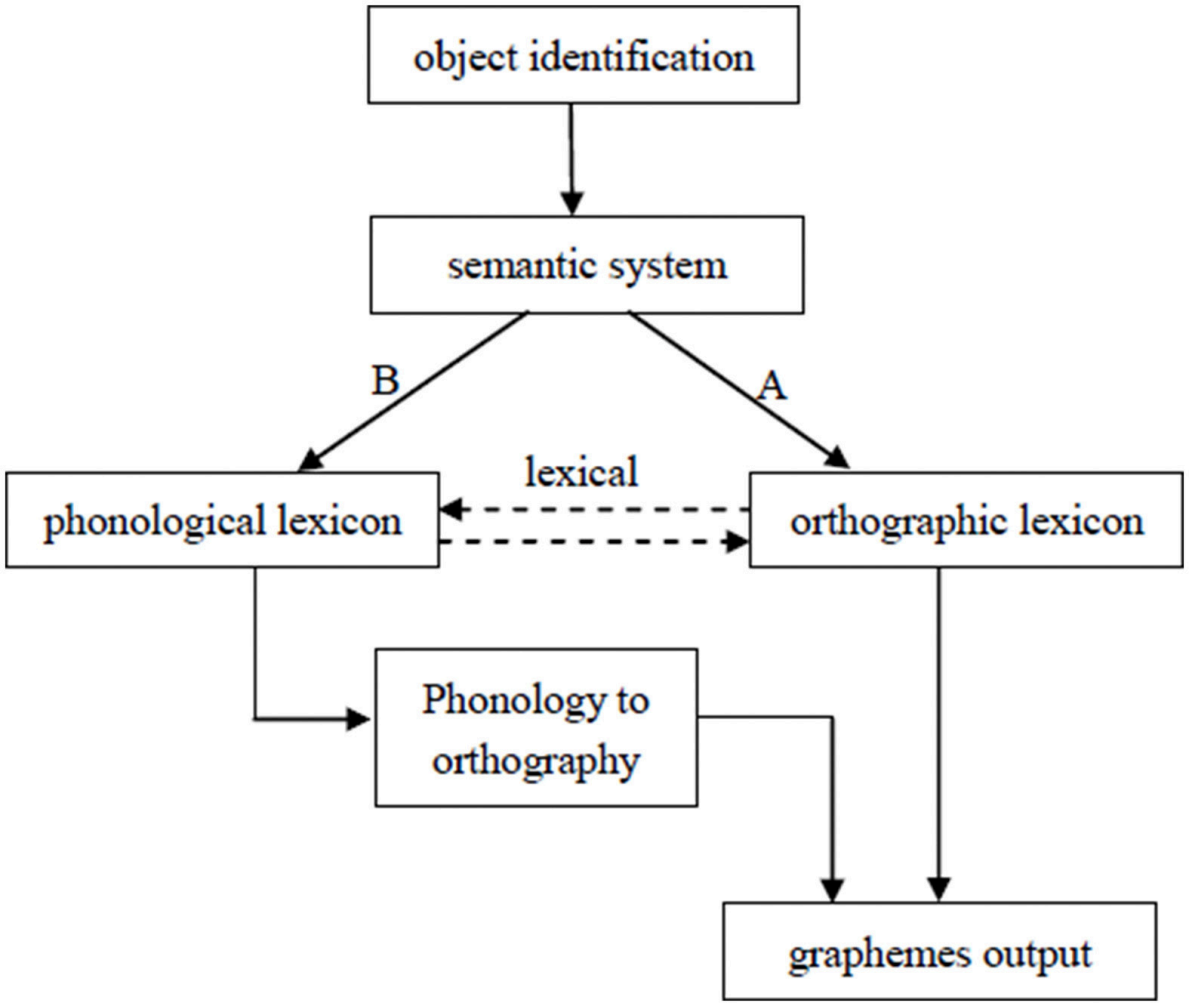
**Sketch model of written picture naming (Bonin et al., 2001b; Damian et al., 2011)**.

Bonin et al. (2001b) propose a working model of written picture naming. The process of written picture naming begins with object identification and conceptual preparation when one is presented a target picture. These representations send activation signals to the orthographic and phonological lexicons in parallel, and there is one-way connection from phonological lexicon to orthographic lexicon, that is, the lexical route. In addition to the lexical route, there also exists a sublexical route that translates phonological representations into graphemic information via phoneme to grapheme conversion. Damian et al. (2011) propose a bidirectional connections between phonological and orthographic lexicons (see also Allport and Funnell, 1981; Patterson and Shewell, 1987). Figure 1 presents a model incorporating the assumptions of Bonin et al. (2001b) and Damian et al. (2011). The orthographic autonomy hypothesis do not deny phonological activation in written production. However, the phonological codes could be activated after orthographic codes by lexical or sublexical links. According to the assumption of the phonological mediation hypothesis, received orthographical codes are activated via phonological codes. That is, the phonological codes are accessed before the orthographic codes, and the properties of the phonological codes affect the retrieval of the orthographic codes.

Though empirical studies have examined the relationship between phonological and orthographic activation using chronometric tasks, the results have been inconsistent. A few studies, however, have found that phonological codes do influence writing (e.g., Bonin et al., 2001b; Zhang and Damian, 2010; Afonso and Álvarez, 2011; Damian et al., 2011; Damian and Qu, 2013; Wang and Zhang, 2015). For example, Zhang and Damian (2010) use a written picture-word interference (PWI) task to examine the role of phonology among English speakers. Distractors were orthographically plus phonologically (OP) related (e.g., picture name: *hand*; distractor*: sand*), orthographically (O) related (e.g., *hand* and *wand*), or unrelated to picture names. An exclusive effect of phonology (OP minus O) at an SOA (stimulus onset aschronyous) of 0 ms and an orthographic priming at an SOA of 100 ms were found, indicating that phonological codes constrain access to orthographic codes at a relatively early stage in written production. Contrary to these findings, Shen et al. (2013) demonstrate a priming effect in English written production due to shared graphemes. This effect is independent of phonological overlapping, thus indicating that handwriting involves complex and abstract graphemic representational units that are in accordance with the orthographic autonomy hypothesis (Rapp et al., 1997).

Evidence of phonological constraints comes largely from studies conducted with alphabetic scripts. This finding is not surprising in the case of alphabetic scripts as phonological and orthographic codes are closely interrelated, and the relationships between phonology and orthography are quasi-systematic in these writing systems. By contrast, orthography and phonology are largely dissociated in Chinese as a non-alphabetic language. It is less obvious why orthographic processing is affected by phonological codes. The orthographic and phonological effects can be separated from each other in such scripts with appropriate manipulation. Using a PWI task, Qu et al. (2011) manipulated the distractors that were OP related (e.g., picture name: 樱桃, /ying1tao2/, meaning *cherry*; distractor: 缨子, /ying1zi/, *tassel*), P related (e.g., picture name: 樱桃; distractor: 英俊, /ying1jun4/, *handsome*), or unrelated to picture names and SOAs (0, 100, and 200 ms). Priming effects were found in the OP-related distractors relative to the unrelated distractors at the 0 and 100 ms SOA, whereas priming in the P-related condition was restricted to the 0 ms SOA. Thus, the findings provide evidence that phonological codes are activated rapidly and that orthographic output is constrained in a non-alphabetic script.

However, there are two potential problems with Qu et al.'s study. First, the degree of phonological overlapping is not matched in the OP-related and the P-related conditions. Most Chinese characters contain a phonetic radical which is a part of a character that indicates how the character as a whole is pronounced. In Chinese script system, the phonetic radical does not always indicate the correct pronunciation of a Chinese character. The phonetic radicals of 15 OP-related distractors and 1 P-related distractor among the 20 presented can indicate the whole character's pronunciation in Qu et al.'s study (see Qu et al.'s material sets for details; e.g., picture name: “樱桃”, /ying1tao2/, *cherry* in English; the OP distractor: “缨子”, /ying1zi/, *tassel*). This may result in a greater facilitation effect in the OP-related condition but a smaller facilitation effect in the P-related condition. Zhao et al. (2012) findings provide evidence for this speculation. A 72 ms phonological facilitation effect at SOA = 0 ms was reduced to 38 ms when phonetic radicals that indicated correct pronunciation were avoided, indicating that when the phonetic radical of a character can indicate the whole character's pronunciation, the magnitude of the phonological effect was affected. Qu et al. observed a significant OP effect (31 ms) and a non-significant P effect (15 ms) at SOA = 0 ms and inferred that the OP effect is orthographic in nature. Due to the aforementioned confounding factor, it is difficult to determine whether the OP effect is orthographic, phonological or a combination of both. Second, Qu et al.'s study did not include the O-related condition, and the authors thus inferred the activation of orthographic codes by comparing the OP effect and P effect.

To avoid these potentials problems, Zhang and Wang (2015) recently conducted a PWI experiment with SOAs of −100 ms, 0 ms and +100 ms. Distractors were OP-related, O-related, P-related or unrelated to picture names. Importantly, the phonetic radicals of the OP-related and P-related distractor words cannot indicate the pronunciation of the characters, and therefore, the potential influence of phonetic radicals is excluded (see Zhang and Wang, 2015 for details). We found an exclusive orthographic effect at an early stage (SOA = −100 ms), reflecting a fast and direct link between meaning and the graphemic lexicon and demonstrating that healthy individuals can access orthographic codes directly from meaning. We also found orthographic and phonological effects at later stages (SOA = 0 ms and +100 ms), reflecting a slow and indirect link between the semantic system and orthographic lexicon via phonology. These findings are not consistent with those from a previous study (Qu et al., 2011), though they do provide evidence in support of the orthography autonomy hypothesis (Rapp et al., 1997).

Although the PWI task with behavioral data offers a typical scenario to tackle the temporal courses of information processing, it can also provide coarse-grained time points of lexical access in spoken and written production. It is well known that the event-related potentials (ERPs) technique can provide high temporal resolution measures. Recently, the combination of the PWI task and ERPs technique has yielded important contributions to understanding the temporal courses of lexical access in speech production (Hirschfeld et al., 2008; Dell'Acqua et al., 2010; Aristei et al., 2011; Piai et al., 2012; Dhooge et al., 2013) and written production (Perret and Laganaro, 2012; Baus et al., 2013; Perret et al., 2014). Perret and Laganaro (2012) compared the common and different ERP waveforms associated with the written and spoken naming of pictures. Similar ERP waveforms correlating speaking and writing appeared until approximately 260 ms after picture onset, reflecting that both spoken and written word production share conceptual and semantic processes, but diverge from word-form (phonological or orthographic) encoding. In a typewriting task, Baus et al. (2013) observe a word frequency effect occurring at approximately 350 ms after picture onset. The word frequency effect, assumed to be an index of lexical access, reflects that speaking and typing diverge from the lexical access stage (including lexical selection and word-form encoding). Perret et al. (2014) manipulate the age of acquisition (AoA) of picture names in picture naming and a writing task while the word frequency remains constant. They report an AoA effect at approximately 400 ms after picture onset in both output modalities, suggesting an orthographic and phonological word from the locus effect with respect to AoA effects (see Bonin et al., 2001a for a behavioral study).

These ERP studies on written production did not address the temporal courses of orthographic and phonological encoding. Therefore, in the present study, we asked participants to perform a PWI task while concurrently recording ERPs time-locked to picture onset. The distractor type (OP-related, O-related, and P-related) and relatedness (related vs. unrelated) between picture names and distractors words were manipulated. Given the high temporal resolution of the ERPs, this experiment allows us to explore the timing at which phonological and orthographic information becomes available and affects writing, thus providing fine-grained time courses of phonological and orthographic activation in written production. Additionally, it allows us to determine whether phonological relatedness interacts with orthographic relatedness. According to the orthography autonomy hypothesis, orthographic codes can be accessed without involving phonological codes. Therefore, we predict that the orthographic effect occurs earlier than the phonological effect and the absence of an interaction between phonological and orthographic effects. According to the phonological medication hypothesis, the retrieval of orthographic codes is dependent on phonological codes, and accordingly, we predict that a phonological effect occurs earlier than the orthographic effect and that there is an interaction between phonological relatedness and orthographic relatedness.

## Methods

### Participants

Twenty-six native speakers of Mandarin Chinese (12 males, age range 19–26 years, mean age 22.7 years) from Renmin University of China and University of Science and Technology Beijing participated in the experiment. Participants were all neurologically healthy, right-handed, and had normal or corrected to normal vision.

### Materials

Fourteen black and white line drawings were selected from a database created by Zhang and Yang (2003). The picture names were monosyllabic words with an average lexical frequency of 0.42 per million (Beijing Language Institute, 1986) and an average stroke number of 10.63. Each target picture was paired with three types of form-related monosyllabic distractor words: (1) an orthographically related (O), but phonologically dissimilar character, that shared the phonetic radical but no syllable with the picture name [e.g., 狐 (fox, /hu2/) - 呱 (crying sound of a child, /gua1/)]; (2) a phonologically related (P), but orthographically dissimilar word, that shared the syllable but no radical with the picture name [e.g., 狐-壶 (pot, /hu2/)]; (3) an orthographically plus phonologically (OP) related word that shared the phonetic radical and syllable with the picture name [e.g., 狐-弧 (arc, /hu2/)]. Regular characters were avoided in all distractors and picture names, excluding the potential phonological influence from the phonetic radicals (see also Zhao et al., 2012). See Appendix A in Supplementary Material for a full list of materials used in the experiment.

The distractors in each condition were then recombined with the picture names to form the corresponding unrelated conditions. Semantic or associative relationships between picture names and distractors were avoided in all combinations. Across three distractor-type conditions, distractor words were statistically matched by the number of strokes and lexical frequency (Beijing Language Institute, 1986), *F*s < 1. See Table 1 for the mean lexical properties of the distractors and Appendix A in Supplementary Material for all of the stimuli used in the experiment.

**Table 1:** **Mean lexical properties of the distractor stimuli used in the experiment**.

	**Distractor type**		
	**OP related**	**O related**	**P related**
Frequency	38.68	65.06	76.76
Number of strokes	9.07	9.5	10.07

Eleven pictures from the same pool were selected as fillers, each of which was paired with three unrelated distractors. These unrelated distractors were also recombined with the filler pictures to form three additional groups of unrelated distractors. Filler and target pictures added up to 25 pictures in total that were evenly distributed into five categories, namely, tools, animals, weapons, musical instruments, and commodities.

### Design

The experimental design included relatedness (related vs. unrelated) and distractor type (O, P, and OP) as within-participants and within-items factors, respectively. For each participant, each picture was displayed under each relatedness condition, and all combinations were repeated three times in separate blocks, resulting in 450 trials. A new pseudorandom sequence was generated for each participant and each repetition, with the constraint that targets or distractors with the same onset did not appear in consecutive trials.

### Apparatus

The experiment was performed using E-Prime Professional Software (Version 1.1; Psychology Software Tools). Participants were seated approximately 70 cm from a CRT monitor with a resolution of 1024 by 768 at 100 Hz. Written responses were recorded with a WACOM Intuos A4 graphic tablet and a WACOM inking digitizer pen (Wacom, Japan).

### Procedure

Participants were tested individually in a sound-proof room. Participants were first asked to familiarize themselves with the experimental stimuli by viewing each picture for 3000 ms with the picture name printed below each picture. Subsequently, they were required to write the picture names and were corrected if necessary. Eight warm-up trials and 150 experimental trials for each block were then administered, with a short break given after 80 trials in each block.

Each trial involved the following sequence. A fixation point (+) was presented in the middle of the screen for 500 ms, followed by a blank screen for 500 ms. A picture and a distractor were then presented simultaneously, and the participants were asked to write the picture name as accurately and quickly as possible while ignoring the distractor. The stimulus disappeared once participants began writing on the graphic tablet or after a time-out of 3000 ms. The next trial began 1500 ms after the experimenter observed that participant had completed writing and was ready for the next trial and, hence, pressed the appropriate key. The experiment lasted for approximately 70 min.

Distractor words were presented in 25-Song font and were centrally superimposed on the target pictures. Pictures were displayed at the bottom of the screen to reduce participants' head and eye movements as they wrote the picture names. Participants were asked to write the picture names on the graphic tablet using an inking pen. During the experiment, participants were instructed to hover the stylus just above the corresponding line on the sheet in anticipation of the response, so that the response would not require arm movement. Participants were also asked to continue gazing the screen and to not monitor their writing (i.e., visual feedback was prevented) to minimize eye and head movement artifacts during the electroencephalogram (EEG) recording (see also, Perret and Laganaro, 2012).

### EEG recordings and analysis

The EEG was recorded via a Neuroscan amplifier (Neuroscan SynAmps) through 66 electrodes located at the standard 10–20 scalp sites secured in an elastic cap (Electro Cap International). The vertical electrooculogram (VEOG) was monitored with electrodes placed above and below the left eye. The horizontal EOG (HEOG) was recorded by a bipolar montage using two electrodes placed on the right and left external cantus. The left mastoid electrode served as a reference. The EEG data were re-referenced off-line to average of both mastoids. All electrode impedances were kept below 5 kΩ. EEG signals were amplified with a band-pass filter of 0.05 and 100 Hz (sampling rate 500 Hz) and were filtered off-line using a 30 Hz low pass filter.

Recordings were analyzed offline using Neuroscan 4.5 software. Prior to off-line averaging, all single-trial waveforms were visually inspected and epochs contaminated by eye movements, electrode drifting, amplifier blocking and EMG artifacts or other noise were rejected. Four participants were excluded from the EEG analysis because of large electrode drift and excessive artifacts. The VEOG electrode activity was applied to ocular artifact correction, and the ocular artifact was conducted using a negative-going EEG at 10% with 40 minimum sweeps at durations of 400 ms. The EEG data were segmented from 200 ms before to 700 ms after the onset of the pictures, with a baseline correction ranging from −200 to 0 ms preceding picture onset. Trials with amplitudes exceeding ±100 μv were eliminated. Only trials with correct responses were considered for ERP analyses.

The amplitudes of the ERP waveforms were analyzed for each distractor type and relatedness condition. The mean amplitude measurements were calculated in three time windows, which were chosen based on the results of an analysis of consecutive 10-ms time windows (see below). Nine electrodes along the sagittal and coronal cerebral axes were selected, namely, the left-anterior (F5), mid-anterior (Fz), right-anterior (F6), left-central (C5), mid-central (Cz), right-central (C6), left-posterior (P5), mid-posterior (Pz), and right-posterior (P6). Repeated measure analysis of variance (ANOVA) was performed on the ERP amplitude means with three factors, specifically, distractor type, relatedness and electrodes, separately in each time window. The Greenhouse-Geisser correction was applied when appropriate. Main effects or interactions involving the distractor type and relatedness that were significant at *p* < 0.05 levels are reported and discussed herein.

In a further analysis, we aimed to identify the temporal onset of orthographic and phonological effects using the jackknife approach (Ulrich and Miller, 2001). At each electrode, different waveforms (related minus unrelated) were generated for each distractor type. A jackknife waveform was then computed for each subject *i* (*i* = 1…*n*, where *n* is the number of subjects) by temporarily omitting subject *i* and computing the grand average of the difference in ERPs from the remaining *n*–1 subject (Ulrich and Miller, 2001). The onset latency was determined by finding the time at which a jackknife waveform reached 50% of the peak amplitude in each time window. The *n* latencies were then averaged at each electrode for each distractor type (Ulrich and Miller, 2001). The ERPLAB plug-in (Lopez-Calderon and Luck, 2014) integrated in the EEGLAB toolbox (Delorme and Makeig, 2004) was used. For each distractor type, the onset latency in a specific time window of the different ERPs was computed at each electrode. Only the latencies at electrodes with significant effects of relatedness were selected, and the smallest of them was used as the final estimate of the onset latency of the effect. To examine whether the latencies of different distractor types were significantly different from each other, the onset latencies of each jackknife waveform were submitted to ANOVA with two within-subject factors, namely, distractor type and electrode. The corrected *F*-value (*F*_*c*_ = *F*/(*n*-1)^2^) and corresponding *p*-value were reported (Ulrich and Miller, 2001).

## Results

### Behavioral results

Trials with incorrect responses (1.86%) and trials with naming onset latencies faster than 300 ms or slower than 2000 ms or deviating beyond 2.5 SD from the cell means (3.63%) were excluded from the analysis. The remaining data were used in the subsequent statistical analyses. Table 2 presents the mean latencies and error percentages as a function of relatedness and distractor type.

**Table 2:** **Mean latencies (M, ms) and percentage of errors (PE, %) as a function of relatedness and distractor type**.

	**O**		**OP**		**P**	
	**M (*SD*)**	**PE**	**M (*SD*)**	**PE**	**M (*SD*)**	**PE**
Related	699 (159)	0.64	698 (179)	1.37	716 (157)	0.92
Unrelated	771 (202)	1.37	769 (183)	1.47	755 (168)	1.01
Effect	72^***^	−0.73	71^***^	−0.10	39^***^	−0.09

*** p < 0.0001.

The data were analyzed using a linear mixed effects model (LMM) (Bates, 2005; Baayen et al., 2008) that included fixed effects of relatedness (related vs. unrelated), distractor type (O, P, OP), by-participant and by-item random intercepts and random adjustments for all fixed effects, according to Barr et al. (2013)'s guideline for the maximal random effects structure. The lmer() function of the lme4 package was used to estimate fixed effects and parameter estimation of the LMM. The degree of freedom and *p*-values were computed using ANOVA() function of the lmer test package with Satterthwaite approximations. These analyses were conducted using the free software R (R Development Core Team, 2013). Latencies and errors were analyzed separately.

For writing latencies, the main effect of distractor type was not significant, *F* < 1, while the main effect of relatedness was significant, *F*_(1, 24.9)_ = 43.41, *p* < 0.0001, as was the interaction between type and relatedness, *F*_(2, 23.0)_ = 5.74, *p* = 0.0.010. Multiple comparisons (*p*-values FDR corrected) revealed that pictures with related distractors were named faster than those with unrelated distractors, this effect was significant for the O distractor type, β = 75.93, *t*_(21.9)_ = 4.87, *p* < 0.0001; for the OP distractor type, β = 78.51, *t*_(19.1)_ = 6.38, *p* < 0.0001; and for the P distractor type, β = 37.91, *t*_(20.0)_ = 5.80, *p* < 0.0001. According to the LMM model, the relatedness effect for the O distractor type (O effect) was comparable to that of the OP distractor type (OP effect), *t* < 1, but was significantly larger than that of the P distractor type (P effect), β = −37.93, *t*_(22.8)_ = −2.45, *p* = 0.023, and the OP effect was also significantly larger than the P effect, β = −40.63, *t*_(24.9)_ = −3.27, *p* = 0.003.

Importantly, we assessed the additivity between orthographic and phonological relatedness with a formula (see Balota and Paul, 1996; Melinger and Abdel Rahman, 2004 for a similar logic) that poses, on the left-hand side, the OP effect (related minus unrelated) and, on the right-hand side, presents the sum of the O and the P effects.

$$\begin{array}{r} \text{OP effect }=\text{ O effect }+\text{ P effect}. \end{array}$$

If the effects of the orthographic and phonological effects are additive, then the two sides of the equation should be statistically equal; if the effects interact, then the two sides of the equation should deviate from zero. For instance, Table 2 indicates an orthographic effect of 72 ms and a phonological effect of 39 ms. An additive relationship would predict an effect of 111 ms (72 ms + 39 ms) for the OP condition. Empirically, the observed OP effect is 71 ms. The difference between the predicted value of 111 ms and empirical value of 71 ms was significant, *t*_(21)_ = 2.41, *p* = 0.025.

Though a parallel analysis was conducted on the errors, a binomial family was used because of the binary nature of the responses. No fixed effect concerning the relatedness or distractor type was significant, |*Z*|s < 1.12, *p*s > 0.26. Adding the distractor type, relatedness, or the interaction between them did not significantly improve the fit, χ^2^s < 12, *p*s > 0.50.

### Electrophysiological results

To avoid contamination of the ERPs due to hand and other muscular movement activity, trials with a writing response faster than 500 ms were removed, and to avoid contamination of extremely slow responses, trials with a writing response slower than 2000 ms were removed. Trials containing artifacts (9.92%) were also excluded in the subsequent analysis.

First, the related and unrelated average waveforms for each distractor type at each electrode were compared via serial paired *t*-tests with a step size of 10 ms in the time-window of 200–500 ms after picture onset. The time intervals were selected as target windows when at least three consecutive *t*-tests approached significance (*p* < 0.05, two tailed, FDR corrected) for each distractor type at one electrode (see also, Li et al., 2008; Zhang and Zhu, 2011). According to this standard, two time windows were selected, specifically, 370–460 and 460–500 ms. The 370–500 ms time window was divided into two because the significance of the phonological effect changed at 460 ms after picture onset.

**Figure 2:**
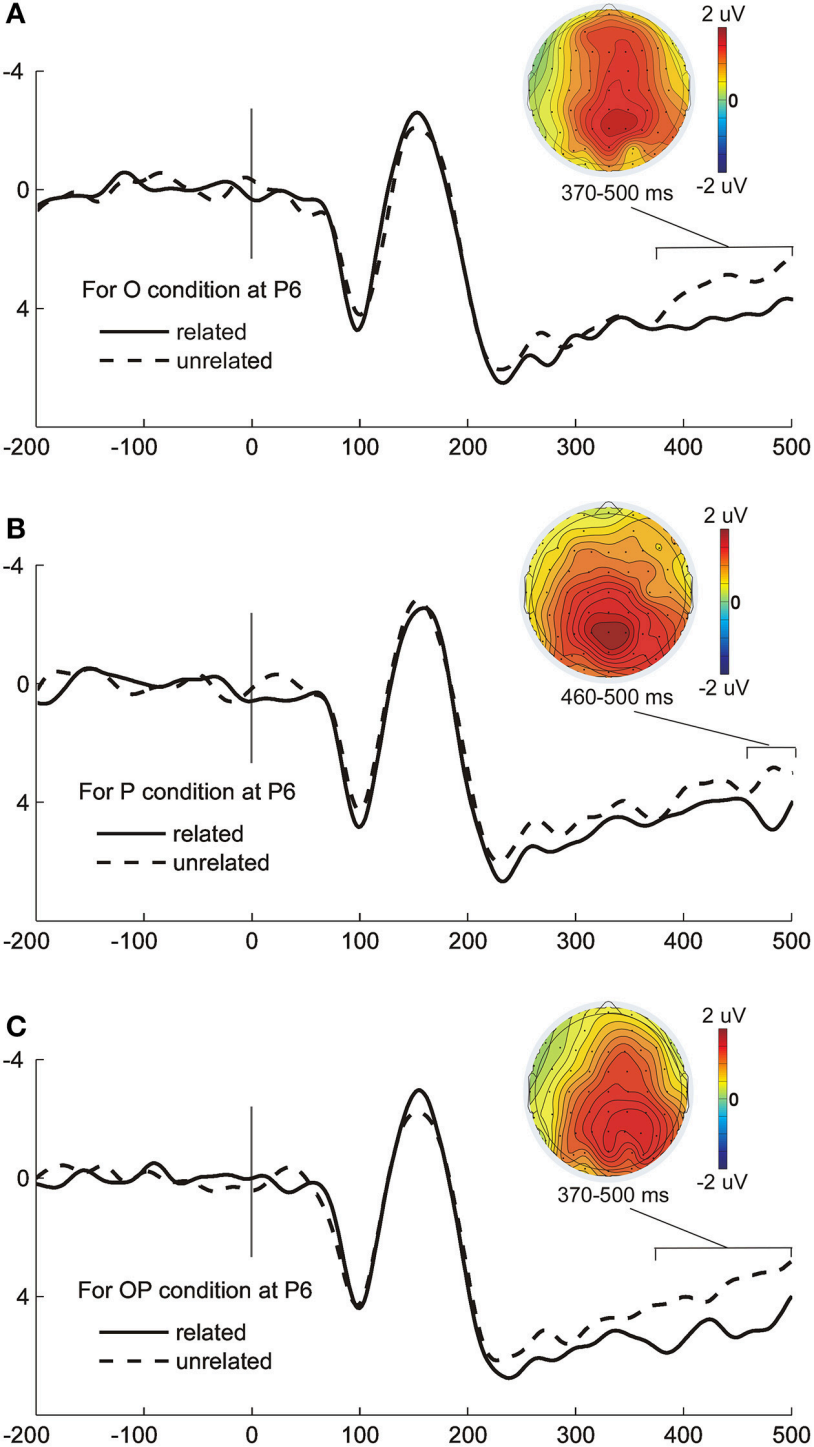
**The grand average ERP waveforms in the related and unrelated conditions for different distractor types and the map distributions for each effect**. **(A)** The ERP waveforms in the orthographically related and unrelated conditions at electrode P6 and the topographical maps of the orthographic effect (unrelated minus related difference) in the time window of 370–500 ms. **(B)** The ERP waveforms in the phonologically related and unrelated conditions at electrode P6 and the topographical map of the phonological effect (unrelated minus related difference) in the time window of 460–500 ms. **(C)** The ERP waveforms in the orthographically plus phonologically related and unrelated conditions at electrode P6 and the topographical map of the phonological effect (unrelated minus related difference) in the time window of 370–500 ms.

Figure 2 presents the grand average ERP waveforms of the related and unrelated conditions for each distractor type and the topographical maps for the O, P, and OP effects (related minus unrelated) in time windows with significant effects of relatedness. The main objective of the experiment was to identify the time signature of the orthographic and phonological effects and to identify a potential interaction between them. To this aim, the amplitude means were analyzed via ANOVAs separately for each time window, with the variables distractor type, relatedness, and electrode as within-subject variables.

In the time window of 370–460 ms, the main effects of the distractor type, *F*_(2, 42)_ = 4.67, *p* = 0.015, and relatedness, *F*_(1, 25)_ = 15.33, *p* = 0.001, were significant. The interaction between electrode and relatedness was significant, *F*_(8, 168)_ = 6.67, *p* = 0.001. Other interactions were not significant, *F*s < 1.76, *p*s > 0.112. Multiple comparisons that assessed the effect of relatedness indicated that the O effect and OP effect were significant in the posterior electrodes (*p*-values FDR corrected, see Table 3). The onset latencies of the O effect and OP effect were 370 and 391 ms, respectively. ANOVA on onset latencies with distractor type (O vs. OP) and electrode as within-subject factors exhibited no significant effects (*F*_*c*_s < 1).

**Table 3:** **Relatedness effect at each electrode for each distractor type in two time windows**.

**Conditions**	**Electrodes**								
	**Frontal**			**Central**			**Posterior**		
	**F5**	**Fz**	**F6**	**C5**	**Cz**	**C6**	**P5**	**Pz**	**P6**
**370–460 ms**									
O effect	—	2.77^†^	—	—	3.44^*^	—	2.29^†^	3.93^*^	3.63^*^
P effect	—	—	—	—	—	—	—	—	—
OP effect	—	—	—	—	2.59^†^	—	2.71^†^	3.09^*^	3.58^*^
**460–500 ms**									
O effect	1.93^†^	2.22^†^	—	1.94^†^	2.80^*^	2.00^†^	2.13^†^	3.41^*^	2.94^*^s
P effect	—	2.01^†^	—	2.32^†^	3.84^**^	2.09^†^	3.42^*^	5.25^***^	4.24^**^
OP effect	2.82^**^	2.35^†^	2.08^†^	2.63^**^	2.80^**^	2.00^†^	2.67^*^	2.97^*^	3.23^*^

† p < 0.10;

* p < 0.05;

** p < 0.01;

*** p < 0.001;

—, non-significance. p-values were FDR corrected.

In the time window of 460–500 ms, the main effects of the distractor type, *F*_(2, 42)_ = 6.46, *p* = 0.004, and relatedness, *F*_(1, 21)_ = 27.07, *p* < 0 0.001, were significant as was the interaction between electrode and relatedness, *F*_(8, 168)_ = 4.97, *p* = 0.004. Other interactions were not significant, *F*s < 0.94, *p*s > 0.46. Multiple comparisons indicate that the O effect, P effect, and OP effect were all significant (*p*-values FDR corrected, see Table 3). The map distributions of the O effect, P effect, and OP effect were similar and mainly distributed in the central and posterior areas. The onset latency of the P effect was 464 ms, and ANOVA on onset latencies with electrode as within-subject factors indicated no significant effect (*F*_*c*_ < 1).

With respect to the additivity between O and P relatedness on the mean amplitudes, we tested whether the formula OP effect = O effect + P effect held true via paired *t*-tests. The results revealed that the OP effect was not significantly different from the sum of the O and P effects at all electrodes in the time windows of 370–460 or 460–500 ms [*t*_(21)*s*_ < 1.16, *p*s > 0.26]. We then conducted a Bayesian analysis using the method suggested by Masson (2011) to test the null hypothesis as well as the BayesFactor package (Rouder et al., 2009) in the R platform. In each time window, the Bayesian factors were greater than three at most electrodes (except one, see Table 4 for details). According to the conventional interpretation of the Bayes factor (3–10, “substantial,” Wetzels et al., 2011), the result suggests substantial support for the null hypothesis (non-interactive) rather than the alternative (interactive).

**Table 4:** **Bayesian factors for the null hypothesis “PO effect = P effect + O effect” of the ERP results**.

**Time windows**	**Electrodes**								
	**F5**	**FZ**	**F6**	**C5**	**CZ**	**C6**	**P5**	**PZ**	**P6**
370–460 ms	3.47	4.13	4.46	4.48	4.25	4.48	2.48	4.37	3.76
460–500 ms	4.46	4.42	4.44	3.94	4.48	4.49	4.41	4.48	3.10

## Discussion

Using a PWI task combined with the ERP technique, we explore the time courses of orthographic and phonological codes and their interplay in written word production. The behavioral results exhibit the typical orthographic and phonological facilitation effects, replicating previous results in Chinese (Qu et al., 2011; Zhang and Wang, 2015). The ERP results reveal that the orthographic relatedness modulates the ERP amplitudes in the time window of 370–500 ms, while the phonological relatedness modulates the ERP amplitudes in the time window of 460–500 ms, reflecting that phonological codes are accessed later than orthographic codes in written production. The jackknife analysis of the onset latency suggests that the orthographic effect and the phonological effect began at 370 and 464 ms after picture onset, respectively. The additivity pattern between orthographic and phonological relatedness was obtained from EEG data. Taken together, our ERP results provide detailed temporal courses of orthographic and phonological encoding in Chinese written production based on the fine-grained temporal resolution. The orthographic codes were accessed earlier than, and independent of, the phonological codes in written production in Chinese. These findings substantially strengthen the existing evidence that orthography is not mediated by phonological information, thus supporting the orthographic autonomy hypothesis (Miceli et al., 1997; Rapp and Caramazza, 1997; Bonin et al., 1998).

### The temporal course of orthographic processing: 370–460 ms

The temporal courses of orthographic processing occur at 370 ms in the O related condition and 391 ms in the OP related condition after picture onset. In the time window of 370–460 ms, the phonological effect was absent and non-interactive between the O relatedness and P relatedness. In addition, the map distributions of both the O effect and OP effect were similar, distributed mainly in the posterior area (see Figure 2). Therefore, we suggest that the OP effect in this time window was orthographic in origin. Moreover, this time point is roughly in accordance with recent studies on written production, that is, approximately 350 ms for a word frequency effect on a typewriting task (Baus et al., 2013) and approximately 400 ms for an AoA effect on a picture writing task (Perret et al., 2014). Both the word frequency effect and AoA effect are typically associated with word-form encoding of the target word (Baus et al., 2013; Perret et al., 2014). By comparing the processes of speaking and writing, Perret and Laganaro (2012) report that the orthographic processing or phonological processing would occur at the stage of word-form encoding, which is 260 ms after picture onset. Thus, we suggest that the orthographic effect should appear during the word-form encoding stage in Chinese written production.

According to Bonin et al.'s (2001b) writing model (see Figure 1), orthographic codes can be accessed by the direct link between the semantic system and orthographic lexicon (arrow A in Figure 1), or they can be converted from phonological codes via phonology-to-orthography mapping at the lexical or sublexical level. Because the results indicate that the orthographic effect occurs earlier than the phonological effect, we suggest that the orthographic effect observed in the present study originates from the semantic representations that spread activation signals directly to the orthographic lexicon. This route has been highlighted in a series of studies (Miceli et al., 1997; Rapp et al., 1997; Alario et al., 2003; Bonin et al., 2015). After approximately 370 ms, written orthographic word forms are accessed and then decomposed into graphemic units (such as graphemes or logographemes) that are stored in the grapheme level and wait to be generated (Caramazza et al., 1987; Han et al., 2007).

One may question the distinct temporal courses of word-form encoding between Perret and Laganaro (2012) and the present study. There were two important divergences between the two studies. The first was the difference in methodology. Where we employed a PWI task with different distractor types to tackle orthographic and phonological processing online, Perret and Laganaro (2012) compare speaking and writing to specify commonalities and differences between the two output modalities. We speculate that the different methodologies have different degrees of sensitivity to the EEG measure, a possibility that must be investigated further. The second was the different target languages. Specifically, Chinese is a non-alphabetic language with in transparent mapping between orthography and phonology, while French is an alphabetic language with relatively transparent mapping between orthography and phonology. The temporal course of word-form encoding is identified in the time window of 250–455 ms in alphabetic languages according to a meta-analysis conducted by Indefrey and Levelt (2004) and Indefrey (2011). By contrast, in an EEG study, Zhu et al. (2015) localized word-form encoding in a time window of 450–600 ms in Chinese spoken word production. Accordingly, the temporal courses of word-form encoding are potentially different between Chinese and French.

### The temporal course of orthographic and phonological processing: 460–500 ms

The O effect and OP effect persisted during this time window. Importantly, the P effect emerged with an onset latency of 464 ms. The O effect, P effect, and OP effect were primarily distributed in the central and posterior areas. Furthermore, there was no interaction between phonological relatedness and orthographical relatedness, suggesting that the OP effect was orthographic plus phonologic in origin during this period. The temporal course of phonological processing, which begins approximately 460 ms after pictures onset, was not consistent with findings for writing (Perret and Laganaro, 2012; Baus et al., 2013; Perret et al., 2014) or speaking (Salmelin et al., 2000; Indefrey and Levelt, 2004; Indefrey, 2011) in alphabetic languages. Salmelin et al. (2000) reported phonological processing in the time window of 200–400 ms in an MEG study. Indefrey and Levelt (2004) estimated that the retrieval of phonological information occurs at approximately 250–330 ms after picture onset. Moreover, this time period was consistent with the findings of phonological encoding (450–600 ms) in Chinese spoken word production (Zhu, et al., 2015).

In the written production framework, we proposed two potential sources for the phonological effect. First, phonological activation could originate from the semantic system. According to the Bonin et al.'s (2001b) model, activation spreads from semantic representations to the phonological and orthographic lexicons in parallel. However, the parallel view of activation would expect similar temporal courses of phonological and orthographic processing, which was contradicted by the present findings. Alternatively, the connections from semantics to orthography were stronger than that from semantics to phonology, and thus, it took less time to retrieve orthographical codes than it did to retrieve phonological codes. The findings with respect to Chinese spoken production provide support for this observation as the magnitude of the orthographic effect was greater than the magnitude of phonological effect, and furthermore, the former occurs earlier than the latter (Bi et al., 2009; Zhang et al., 2009; Zhang and Weekes, 2009). By contrast, Zhao et al. (2012) noted that orthographic and phonological facilitation effects were of equal size and had very similar time courses. Thus far, findings on writing and speaking in Chinese suggest that the orthographic codes were not accessed later than the phonological codes regardless of the output modality.

Second, there are bidirectional connections between the phonological and orthographic lexicons (Damian, et al., 2011). Orthographic codes are activated at the semantic level and then spread to the phonological codes, which results in a phonological facilitation effect. Furthermore, there was a 94 ms interval between the onset latencies of the orthographic effect (370 ms) and phonological effect (464 ms), suggesting that it may take approximately 90 ms for activation to spread from the orthographic lexicon to the phonological lexicon. There was an overlapping time period for orthographic and phonological activation after 460 ms, which was in accordance with the cascaded pattern between the orthographic and phonological levels in the written production system. Qu and Damian (2015) suggest a similar pattern between the semantic and orthographic levels. These findings suggest that the cascaded view of activation could be a central principle in written production as well as in spoken production.

Taken together, the ERP results reveal that orthographic codes are accessed earlier than phonological codes, which is consistent with a behavioral study (Zhang and Wang, 2015) of varied SOAs in a PWI task (see the introduction for details). Several studies on alphabetic languages (e.g., French and English) also found that phonological overlapping did not affect orthographic output in writing (Bonin et al., 1998; Roux and Bonin, 2012; Shen et al., 2013). Additionally, the absence of the interaction between the O-related and P-related effects implies that orthographic codes and phonological codes could be accessed independently. Several neuropsychological studies have demonstrated successful writing in the face of damaged phonological retrieval or double dissociation between written and oral picture naming (Bub and Kertesz, 1982; Miceli et al., 1997; Rapp et al., 1997; Alario et al., 2003).

Several other studies have demonstrated that phonological factors influence writing in English (Damian et al., 2011; Damian and Qu, 2013), French (Bonin et al., 2001b; Afonso and Álvarez, 2011) and Chinese (Zhang and Wang, 2014; Wang and Zhang, 2015). For instance, Zhang and Wang (2014) manipulated the word frequency and the syllable frequency of picture names. Pictures with a high syllable frequency were written faster than those with a low syllable frequency, thus indicating that the phonological properties may constrain the generation of orthographic output codes. However, these findings of a phonological effect do not contradict the orthographic autonomy hypothesis because this hypothesis assumes independent orthographic activation and does not deny phonological activation in writing (Miceli et al., 1997). Thus, it is concluded that the underlying mechanisms of the phonological role in writing requires further investigation.

The pattern of the interplay between orthographic and phonological relatedness was found to be interactive in the behavioral data but additive in the EEG data. The EEG data reflect online planning processing before 500 ms, while the behavioral data indicate that all processes are involved in writing before execution. In the present study, we exclude data with naming latencies faster than 500 ms or slower than 2000 ms. The average writing latencies were approximately 735 ms. We speculate that the interaction between orthographic and phonological relatedness occur after 500 ms, when both orthographic and phonological codes enter into the production system. Zhang et al. (2009) found that in a PWI task with various SOAs, the additive pattern between orthographic and phonological relatedness is evident at SOAs of −150 ms and 0 ms, but an interactive pattern is evident at an SOA of +150 ms in Chinese spoken production. By manipulating the entry time of the distractor information relative to the onset of the picture, we can tap into the early stage at SOAs of −150 ms (distractor onset before picture) or 0 ms, as well as the relative late stage at an SOA of 150 ms (distractor onset after picture). This finding suggests an additive pattern at the lexical level, but a non-additive pattern at the post-lexical level and thus provides support for our speculation regarding the dissociation between the behavioral and EEG data.

What are the implications of our findings for written production? In Bonin et al.'s model for written production, our finding of an early processing stage in which priming is dominated by orthographic relatedness suggests that activation occurs quickly and directly from the semantic system to the graphemic codes. By contrast, the link from semantic representation to phonological codes may be relatively weak. The orthographic effect (370–500 ms) was approximately 90 ms earlier than the phonological effect (460–500 ms), indicating that it might take approximately 90 ms to transition from the orthographic lexicon to the phonological lexicon. Chinese, as previously mentioned, is a language in which activation of phonology and orthography could be separated via appropriate manipulation, thereby providing a good opportunity to investigate orthography with little phonological confounding. Thus, the results of the present study provide compelling evidence for the hypothesis that orthographic activation does not depend on prior phonological activation. These results are not unusual given the Chinese written language. First, there is no transparent sublexical phonology to orthography correspondence in Chinese characters, and any activation from phonology to orthography would be slow. Second, homophones are abundant in Chinese (Zhou and Shu, 2008), and there are multiple characters corresponding to one pronunciation, which results in an inefficient route to access orthographic codes through this connection.

In conclusion, we found that orthographic codes were accessed earlier than, and independent of, phonological codes in the written production of Chinese. The results are interesting because they provide detailed temporal courses of orthographic and phonological activation in Chinese written production. These findings substantially strengthen the existing evidence that orthography is not mediated by phonological information, and they provide important confirmation of early orthographic influences on written production, which is in accordance with the orthographic autonomy hypothesis rather than the phonological mediation hypothesis.

## Ethics committee

The current study was approved by the Independent Ethics Committee of the Institute of Psychology, Chinese Academy of Sciences in Beijing.

## Author contributions

QZ, CW designed the experiment and prepared the manuscript. CW conducted the experiment and analyzed the data.

## Funding

This research was supported by grants from the National Natural Science Foundation of China (31170977, 31471074), the fundamental research funds for the Central Universities, and the Research Funds of Renmin University of China (No. 14NXLF12) to QZ.

### Conflict of interest statement

The authors declare that the research was conducted in the absence of any commercial or financial relationships that could be construed as a potential conflict of interest.
