# Supplementary material for: The Temporal Courses of Phonological and Orthographic Encoding in Handwritten Production in Chinese: An ERP Study
Source: Front Hum Neurosci. 2016 Aug 24;10:417. doi: 10.3389/fnhum.2016.00417 (PMC4995206; doi:10.3389/fnhum.2016.00417)
Supplement: Supplementary file 1 [file Presentation1.PDF]

Appendix A. Materials used in the experiment

picture names	Distractors		
	The OP related	The O related	The P related
猪(pic,/zhu1/)	诸(various,/zhu1/)	绪(beginning,/xu4/)	株(a plant,/zhu1/)
狐(fox,/hu2/)	弧(arc,/hu2/)	呱(crying of baby,/gua1/)	壶(jug,/hu2/)
鸭(duck,/ya1/)	押 detain,/ya1/)	钾(kalium,/jia3/)	丫(forked,/ya1/)
箭(arrow,/jian4/)	煎(fry,/jian1/)	俞(a surname,/yu2/)	兼(and,/jian1/)
梳(comb,/shu1/)	疏(to dredge,/shu1/)	琉(sparkling stone,/liu2/)	殊(special,/shu1/)
瓶(bottle,/ping2/)	屏(screen,/ping2/)	骈(parallel,/pian2/)	凭(rely on,/ping2/)
耙(rake,/pa2/)	杷(loquat,/pa2/)	肥(fat,/fei2/)	爬(crawl,/pa2/)
桶(bucket,/tong3/)	捅(stab,/tong3/)	涌(gush,/yong3/)	筒(canister,/tong3/)
蛙(frog,/wa1/)	洼(swamp,/wa1/)	鞋(shoe,/xie2/)	挖(dig,/wa1/)
笛(flute,/di2/)	迪(follow,/di2/)	抽(extract,/chou1/)	涤(wash,/di2/)
鹅(goose,/e2/)	俄(Russian,/e2/)	哦(oh,/o4/)	额(forehead,/e2/)
锅(pan,/guo1/)	埚(crucible,/guo1/)	娲(a goddess,/wa1/)	郭(a surname,/guo1/)
剑(sword,/jian4/)	俭(frugal,/jian3/)	脸(face,/lian3/)	件(piece,/jian4/)
枪(gun,/qiang1/)	呛(choke,/qiang1/)	怆(sorrow,/chuang4/)	腔(cavity,/qiang1/)

Note: OP-Orthographically plus phonologically, O-Orthographically, P-Phonologically.
